# VIEWS ON AGING AND PERCEIVED DISCRIMINATION: THE MODERATING ROLE OF GENDER TYPICALITY

**DOI:** 10.1093/geroni/igae098.3766

**Published:** 2024-12-31

**Authors:** Kelly Smith, JoNell Strough

**Affiliations:** West Virginia University, Morgantown, West Virginia, United States; West Virginia University, Morgantown, West Virginia, United States

## Abstract

Age and gender are outwardly visible personal characteristics upon which people make social judgments (Cuddy & Fiske, 2002). As such, these different characteristics may be associated with perceived discrimination. Here, we examined whether perceived discrimination varied depending on individuals’ views of their own aging (VoA) and their gender typicality when each was measured as a multidimensional construct. VoA was comprised of aging-related cognitions, self-perceptions of aging, and subjective age. Gender typicality was comprised of gender roles and expectations, gender self-concept, and gender social presentation. Participants (N = 616; 40-93 yrs., M = 53.43 yrs.; 56% women, 85% White) completed self-report measures collected online via Prolific. Interactions between VoA and gender typicality were assessed using an SEM framework in Mplus, and there were two significant interactions among components of VoA and gender typicality. First, perceptions of discrimination were greater for participants who reported less positive aging-related cognitions and highly-gender-typical gender roles and expectations. Second, perceptions of discrimination were greater for those with less positive self-perceptions of aging, and this was amplified for those with lower gender typicality (roles and expectations). Together, these findings suggest that high and low gender typicality in the form of conformity to gender roles and expectations are differentially associated with perceptions of discrimination depending on VoA. The importance of considering multiple personal characteristics to better understand perceptions of discrimination is discussed along with recommendations for future research that considers the intersection of VoA and other individual difference characteristics.

